# A novel approach for estimating fertility rates in finite populations using count regression models

**DOI:** 10.1038/s41598-024-51734-z

**Published:** 2024-01-22

**Authors:** Shakeel Ahmed, Olayan Albalawi, Javid Shabbir

**Affiliations:** 1 School of Natural Sciences, NUST, H-12 Islamabad, 44000 Pakistan; 2 Department of Statistics , Faculty of Science, University of Tabuk, Tabuk, 47512 Saudi Arabia; 3 Department of Statistics, University of Wah, Wah Cantt, Pakistan

**Keywords:** Health care, Mathematics and computing

## Abstract

Demographic health surveys (DHS) contain in-depth information about the demographic characteristics and the factors affecting them. However, fertility rates which are the important indicators of population growth have been estimated by utilizing the design-based approaches. Model-based approach, on the other hand, facilitates efficient predictive estimates for these rates by utilizing the demographic and other family planning related characters. In this article, we first attempt to observe the effect of various socio-demographic and family planing related factors on births counts by fitting different regression models to Pakistan Demographic Health Survey 2017–2018 data under classical as well as Bayesian frameworks. The births occurred during the time periods of 1-year, 3-years and 5-years are taken as the responses and modeled using different non-linear models. The model-based approach is then used for estimation of the fertility measures including age-specific fertility rates, total fertility rate, general fertility rate, and gross reproduction rate for ever-married women in Pakistan. The performance of the model-based estimators is examined using a bootstrapped sampling algorithm. While the age-specific fertility rates are over-estimated for some age groups and under-estimated for others. The model-based fertility estimates are recommended for estimating the demographic indicators at national and sub-national levels when survey data contains incomplete or missing responses.

## Introduction

Measuring fertility indicators using household surveys such as the Demographic Health Surveys (DHS) and the Multiple indicator Cluster Surveys (MICS) is challenging especially in developing countries like Pakistan, where updated vital registration systems (VRS) are very poor or are not available at all. Such measures are needed to evaluate the progress of the United Nations (UN) Sustainable Development Goals (SDGs), which is especially to ”*Ensure healthy lives and promote well-being for all at all ages* ”, for the improvement of maternal, newborn, and child health^1,2^. Fertility measures as an important indicator of the above stated SDG needs to be estimated for evaluation of the progress on SDGs. The direct fertility indicators estimation methods were initially utilized by the practitioners in World Fertility Survey (WFS), which was conducted during 1972–1984^3,4^, and afterward in the DHS surveys. The approaches utilized by WFS and DHS have been documented in many articles like^5–7^. The approaches have later been used for estimation of fertility indicators by other household surveys (HS) programs, such as the MICS. In direct estimation methods, data about the births occurred during 3 or 5 years are gathered and used for the calculation of fertility indicators^8^. The DHS program mostly uses the census and survey processing system and other typical statistical packages such as STATA, SAS, SPSS, or R, etc. to produce the fertility measures, and other DHS statistics under direct methods^9–11^. The results on fertility indicators obtained from PDHS 2017–2018 data, using DHS.rates package^12^ (constructed by^13^), are given in PDHS 2017–2018 report^14^. The report covers a cross tabulation and visual display of the relationship between the fertility indicators and other demographic and socio-economic factors (Region, Sex, Marital Status, Age, Education, etc). Apart from reaching to a valid statistical inference, estimation of parameters, and constructing confidence intervals based on estimated rates, one can also develop regression models for the number of births and estimate them to observe the relationship between the number of births and its candidate determinants^15^.

Etiological studies utilize the regression models for establishing cause and effect relationship between number of births and its determinants. However, the births history includes discrete variables where the linear models cannot be applied as the normality of residual errors does not fulfill^16,17^. Generalized Linear Models (GLMs) are fitted for prediction in situations where outcome variables have a distribution other than normal. Because GLMs involve categorical variables of interest such as “Yes”/“No” responses; or belonging to Groups A or B and, therefore, do not have full range i.e. $-\infty$ to $+\infty$. Hence, the relationship between the response variable and the predictors may not be linear. Some examples of GLMs used for birth history data include logistic regression model^18^, Poisson regression model^19^ and event history methods^20^. Although the principle has rarely been described in demographic analysis manuals, regression methods, especially Poisson regression, can still be practiced to calculate the classical demographic measures such as total fertility rate (TFR) and other fertility rates^21,22^. Some new developments and applications on fertility estimation and casual inference are available in^23–28^. Reference^29^ used 1998–1999 Burkina Faso DHS data to explain the estimation of TFR and ASFRs using individual data with Poisson regression. The number of births over a 5-years period preceding the interview (variable predefined in the DHS women recode file PKIR71) is taken as the response variable and the age groups as dummy regressors in the model controlling for the length of exposure (5-years corresponding to each woman) using a term (offset)^30,31^. The average number of births is obtained by exponentiating the coefficients for each of the seven age groups without introducing an intercept and computing the TFR as the sum of the rates multiplied by five. Recently^32^, obtained pooled estimate of the TFR in sub-Saharan Africa using (2010–2018) DHS data.

The classical linear regression provides estimate of the model parameters based on the sampled data alone. However, if the sample size is small, one might express the estimate as a distribution of possible values of the parameter given the sample information. This is the situation where Bayesian regression is needed. Bayesian regression is widely used in estimation, inference and prediction purpose in wide variety of non-linear setups^33,34^. In Bayesian regression parameter estimation is done using Markov Chain Monte Carlo (MCMC) method^35^. Reference^35^ warned about the programming errors and the problems that occur in estimation routines during MCMC. However, Bayesian framework is still preferred due to the flexibility in model construction, statistical inference, and assessment of the fitted model than the classical approaches^36^. Reference^37^ also pointed out two main concerns with the employment of the MCMC algorithms: mixing and convergence and suggested to confirm that the algorithm results in a Markov chain that “converges” to the appropriate posterior density and “mixes” well throughout the values of the density. When these conditions met, Bayesian regression nicely models the linear as well as non linear relationship between the response variable and covariate with an effective prediction^38,39^.

**Figure 1:**
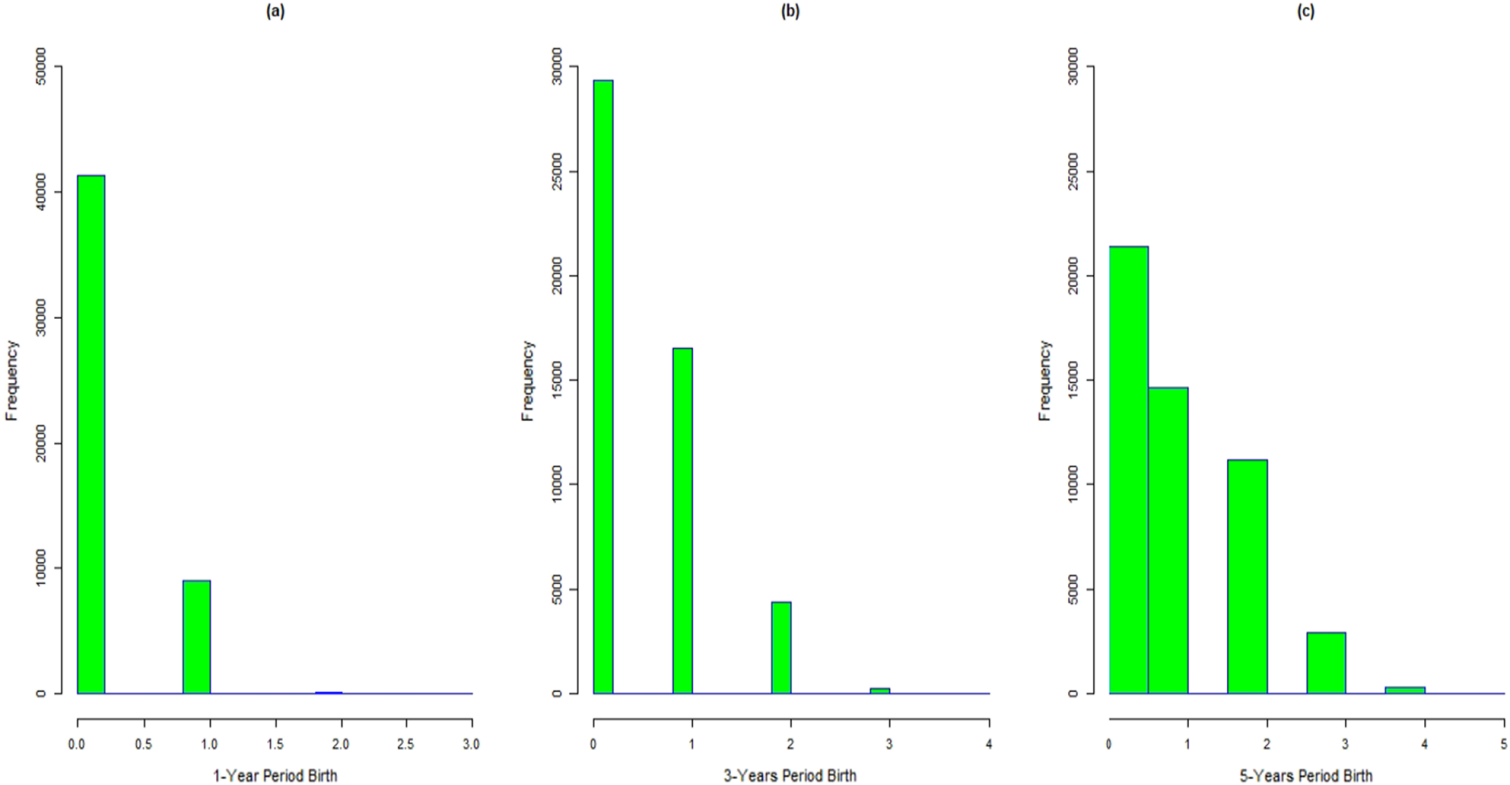
(**a**) Bar chart for birth counts during 1-year period. (**b**) Bar chart for birth counts during 3-year period. (**c**) Bar chart for birth counts during 5-years period; X-axis consists of birth counts and Y-axis corresponding frequencies.

The literature on fertility estimation emphasizes on observing the relationship between the birth counts and their determinants as well as utilizing the estimated models for obtaining more accurate estimates at national and sub-national levels. However, the model-based predictive estimation method aids in efficiency by utilizing the model relationship between the study variable and the available socio-demographic variables^40,41^. However, the response variables are the birth counts which, we cannot proceed with linear regression setup. For obtaining a precise estimates of fertility measures, we establish the model-based estimators of fertility measures using the classical as well as the Bayesian count regression models. The model-based approach increase efficiency of the estimators by including information available on related covariates. We are able to produce estimates on different fertility measures with age sex distribution at national as well as sub-national levels for fixed values of covariates. Based on the suggested estimation algorithm we can produce fertility estimates for fixed values of other significant socio-demographic and family planning variables. For example, we can produce estimates on fertility indicators based on Wealth Index (WI), education level of women, contraceptive usage etc. An etiological analysis on number of birth is conducted in Section “Model estimation and inference for birth counts”. Section “Model-Based Estimation of Fertility Indicators” covers the model-based estimation of different fertility rates. Section “Conclusion” summarizes the article with some concluding remarks (Fig. 1).

## Model estimation and inference for birth counts

We first briefly introduce fertility indicators and methods of their calculation following^5^. The Age Specific Fertility Rate (ASFR) is the average count of births that occurred during a given reference period per 1,000 women exposed to the risk of fertility, in 1- year, 3-years, or 5-years age groups. For any age group $g= 1, 2,...,7$ for 5-years grouping), $B_{g}$, and $E_g$ denote the total births given by the women and women-years of exposure in age group *g* for the referenced period respectively. The ASFR in age group *g* is expressed as:

1 $$\begin{aligned} ASFR_{g}=\frac{B_{g}}{E_{g}} \times 1000, \end{aligned}$$

where 1000 is multiplied to show the rate per 1000 women-years of exposure. The data about the exact Date of Birth (DoB) of the child from DHS data was utilized for directly calculating the numerator $B_{g}$. For calculating the denominator $E_g$, the exact DoB of each woman was used for summing up the number of women-years of exposure in *g*, because a woman can participate in atleast two age groups for a given referenced period. For details about computation of the women-years of exposure, readers are referred to^11^. The Total Fertility Rate (TFR) is used to measure women’s fertility hypothetically^42^. It can be described as the total children who would be born per 1,000 women if they went through their reproductive age according to a given schedule of ASFR subject to no mortality. The TFR is computed on the basis of $ASFR_{g}$ for $g=0,1,...,6$ as follows

2 $$\begin{aligned} TFR=5 \times \sum _{g=0}^{6}ASFR_{g}. \end{aligned}$$

The General Fertility Rate (GFR), which is the mean count of births that a woman gives during her whole reproductive period, is obtained by dividing the total number of births during a specified period by the total number of women exposed to the risk of fertility, during the same specific period. The standard formula for GFR is given by

3 $$\begin{aligned} GFR=\frac{\sum _{g=1}^{7}B_{g}}{\sum _{g=1}^{7}E_{g}}. \end{aligned}$$

**Figure 2:**
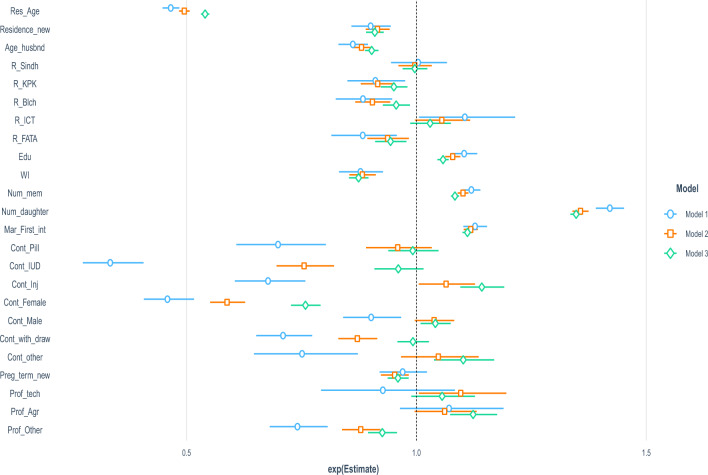
Display of exponentiated estimated coefficients for Poisson models.

Similarly, Gross Reproduction Rate (GRR) is computed based on ASFR and sex ratio at birth during that period. Like TFR, GRR assumes that the hypothetical group (cohort) of women pass from birth over their reproductive age with no mortality. This assumption is valid when one is interested in comparing levels of fertility over time. The GRR is expressed as

4 $$\begin{aligned} GRR_{g}=5 \times \sum _{g=0}^{6}ASFR_{g}\times P_{g}, \end{aligned}$$

where $P_{g}$ is the proportion of female births to the women in age group *g*. The birth data file (PKBR71FL.DTA) from PDHS 2017-18 was taken for analysis. The detail about the data collection mechanism, fieldwork, training of staff and pretest is available in^43^. The key variables including demographic characteristics, socioeconomic variables, and variables related to family planning were taken as the predictors and described in Table 1 along with the response variables. Figure 2 shows bar-charts corresponding to three responses and one can observe that all three responses depict highly departure from symmetry. Also one can observe that the number of births for 1 year includes the highest number of zeros than the other two counts. The number of births during the 5-year period tends to follow a Poisson distribution without access to zeros.

**Table 1:** Variable Description.

Variables ID	Variable name	Variable description
v024	Region	Region: 1 = Punjab, 2 = Sindh, 3 = Khyber-pakhtun-khwah (KPK),
		4 = Balochistan, 5 = Gilgit Baltistan (GB), 6 = Islamabad Capital
		Territory (ICT), 7 = Azad Jammu and Kashmir (AJK),
		8 = Ex-Federally administered tribal areas (FATA)
	Sindh	Dummy variable: 0 = Other Regions, 1 = Sindh
	KPK	Dummy variable: 0 = Other Regions, 1 = KPK
	Balochistan	Dummy variable: 0 = Other Regions, 1 = Balochistan
	ICT	Dummy variable: 0 = Other Regions, 1 = ICT
	FATA	Dummy variable: 0 = Other Regions, 1=FATA
v025	Residence	Dummy variable: 0 = Rural, 1 = Urban
v106	Edu	Highest educational: 0 = No education at all, 1 = Attend primary school,
		2=Attended Secondary School, 3 = Higher Education
v191	WI	Dummy variable: 0 = -ve Wealth index, 1 = +ve Wealth index
		i.e. 1 v191>0,0 otherwise
v201	Num_mem	Total children ever born 0:20
v203	Num_daughter	Number of daughters at home 0:20
v221	MTFBI	Marriage to first birth interval (MTFBI) (months) 0:350
		0=when v221=996
		and Negative interval (i.e. birth before marriage)
v312	Cont_Pill	Contraceptive pill usage: 1 = Pill, 0 = otherwise
	IUD	Intrauterine device usage: 1 = IUD, 0 = otherwise
	Cont_Inj	Contraceptive injection usage: 1 = Injections, 0 = otherwise
	Cont_female	Female contraceptive: 1 = Female sterilization, 0 = otherwise
	Cont_male	Male contraceptive: 1 = Male sterilization and Male condom, 0 = otherwise
	Cont_withdrawal	Contraceptive withdrawal: 1 = Withdrawal, 0 = otherwise
	Cont_other	Dummary corresponding to remaining categories leaving
		“No Using” as base category
v239	Preg_term Content$^{1}$	Pregnancies terminated before calendar beginning
		0 = No 1 = Yes (m) 9 = Missing
v717	Prof_tech	Technical profession: 1 = Professional/technical/managerial
		or Clerical, 0 = otherwise
	Prof_Agr	Agriculture profession: 1 = Agricultural - self employed, 0 = otherwise
	Prof_Other	Dummy corresponding to remaining categories taking
		“Not working as base”
v730	Age_husbnd$^{2}$	Husband/partner’s age 15:94
v209	Birth_1y	Births in 1 year preceding the interview 0:4
v238	Birth_3y	Births in 3 years preceding the interview 0:4
v208	Birth_5y	Births in 5 years preceding the interview 0:6

$^{1}$ The values of Preg_term are reported after imputing 0 in missing cases within first 13,123 and 1 in remaining 37,373 cases. Note that this division is made by dividing the data in ratio $4676\times (50495)/(13317+4676)$ and $13317 \times (50495)/(13317+4676)$.

$^{2}$ Missing entries in Age_husbnd was imputed by median age of the observed responses.

### Birth count regression models

Let *y* be the observed response corresponding to a random variable *Y* whose values are unknown for a finite population of size *N* indexed as $\mathcal {U}=\{1,2,3,....,N\}$. In matrix notation, let $\varvec{y}=(y_{i}, i \in U)$ be the realizations of the stochastic vector $\varvec{Y}=(\varvec{Y}_{i}, i \in \mathcal {U} )$ under model-based approach. Suppose a sample $S=\{1,2,3,...,n\}$ of size *n* is drawn from finite population $\mathcal {U}$ using a Sampling Design (SD) and $r=(1,2,3,...,N-n)$ be the set of index attached to the values of units that are not indexed in *s*. For a given sample *s*, we can rearrange the population vector as $\varvec{y}=(\varvec{y}^{T}_{s},\varvec{y}^{T}_{r})^{T}$, where $\varvec{y}_{s}$ and $\varvec{y}_{r}$ be the vectors of *n* sampled and $(N-n)$ non-sampled values of the study variable respectively. Let $\mathcal {M}$ be the true underlying model expressed as

5 $$\begin{aligned} \varvec{Y}= \varvec{X}\varvec{\beta }+\varvec{\epsilon }, \end{aligned}$$

where $\varvec{X}$ is the data matrix containing *p* regressors including intercept and $\varvec{\epsilon }$ be the vector of random errors assumed to be distributed normally with mean vector $\varvec{0}$ and variance-covariance matrix $\varvec{\Sigma }$. When the response variable is the number of occurrences of an event, the distribution of counts is discrete and is bound to non-negative integered values. While applying an ordinary linear regression model to such data, researchers may face one of the following two issues. (i) Often such count data has positively skewed distribution with many observations having value 0 as in Fig. 2. With a large number of zeros in the data set, one cannot transform such skewed distributions into normal. (ii) It is quite possible that the regression model produces negative predicted values which contradicts with theory^44^. The following sub-sections cover some generalized linear regression models which we use as a working model for births per woman during 1, 3, and 5-years periods before the interview.

#### Poisson regression model

A Poisson Regression Model (PRM) assumes that the error term has Poisson distribution instead of a normal distribution. Further, it uses the natural logarithm of the response variable as a linear function of the coefficients rather than simply modeling the response variable as a linear function of the regression coefficients. To proceed it is assumed that the logarithm of the mean values (rates) can be modeled into a linear form with some unknown parameters. The mathematical form of PRM is given by

6 $$\begin{aligned} {\textbf {log}}\big (\varvec{y}\big )=\varvec{X}\varvec{\beta }, \end{aligned}$$

where vector $\varvec{\beta }$ is obtained using maximum likelihood estimation (MLE). Let $\mu$ be the rate parameter which is also the dispersion parameter of Poisson distribution and Equation (5) can be expressed as:

7 $$\begin{aligned} {\textbf {log}}\big (\varvec{\mu }\big )= \varvec{X}\varvec{\beta }. \end{aligned}$$

The exponent of the coefficient $\beta _{j}$ (*j*th component of $\varvec{\beta }$ for $j=0,1,2,...,p$) for an explanatory variable $(X_{j})$ thus shows the relationship between the number of births per woman for which the explanatory variable has a specified value and the number of births per woman for which the variable has the specified value minus one, all other things remain constant. Readers can find more details about Poisson regression model from^45^.

#### Negative binomial regression model

The PRM assumes that the error term, consequently responses for fixed covariate values, has the same mean and variance which is not usually true in practice. In many cases we face problem of over dispersion i.e. the variance of the error is larger than its mean. An alternative method for modeling the data with an over-dispersed error term is to fit a Negative Binomial Regression Model (NBRM)^46^. In negative binomial distribution the parameter of the distribution is considered as a random variable. The variation in the parameter can be considered as the variance of the data that is larger than the mean. We need more parameterization in NB distribution to get a form that is appropriate to our model. Following the notations given in^47^, we parameterize the NB density for the *i*th observation with parameters $P_{i}$ and *r*. The former is known as the success parameter, and for the *i*th observation it is defined as $P_{i}=\frac{r}{r+\mu _{i}}$, where $\mu _{i}$ satisfies the relation given in Equation (7). The latter is the over-dispersion parameter $(\ge 0)$, which is equal to 1 in the Poisson distribution (i.e. there is no over-dispersion). The maximum likelihood estimates of the coefficients are obtained using the MASS package in R. The detail about parameter estimation, model assumptions, and validity of estimates can be found in^48^, Page 326 and^49^.

#### Zero-inflated poisson model

In the cases with responses having a large variance, many zeros as well as a few very large values, the negative binomial model as an extension of Poisson handles the extra variance. However, sometimes there may exist too many zeros than a Poisson would expect to predict. In such cases, a better option is to use Zero-Inflated Poisson (ZIP) model^50^. In a ZIP model, we assume that some zeros occur by a Poisson process and some were not even able to have the event occur. Hence two processes work in ZIP – where one determines whether the individual is eligible for a non-zero response, and the other finds the count of that response for eligible individuals. The ZIP model consists of two regression models both working simultaneously. A logistic (or probit) model is used to determine the probability of being eligible for a positive count and a Poisson model is used to model the size of the counts for eligible individuals with positive value. Both models utilize the same predictors, but with separate estimates for their coefficients. In this way, the predictor variables can have quite different effects on the processes. While a ZIP model needs it to be theoretically reasonable that some individuals are not eligible for a count. Zero-Inflation Poisson (ZIP) for response *y* is defined as

$$\begin{aligned} P(y)= {\left\{ \begin{array}{ll} \theta +(1-\theta )\text {Pois}(0|\mu ), &{}\text {if }y = 0;\\ (1-\theta )\text {Pois}(y|\mu ), &{}\text {if }y \ge 0 \end{array}\right. } \end{aligned}$$

where $$\theta $$ is the probability of occurring false values (zeros). Hence there are two models coupled together (a mixture model) to give an overall probability: (1)-when a response is zero (i.e. $$ y_{i}=0$$), it is the probability of getting a zero plus the probability of a true value times probability of choosing a value of zero from a Poisson distribution with parameter $$\mu $$ and (2)-when a response is greater than 0, it is the probability of true value times the probability of drawing that value from a Poisson distribution with parameter $$\mu $$.

This definition indicates that the Poisson parameter $$\mu $$ is the same for both zero and non-zero components. The model of zero values (i.e. $$ y_{i}=0$$) is used for essentially investigating whether the likelihood of false zeros is related to the linear predictors. The greater than zero (i.e. $$ y_{i}\ge 0$$) model, then, investigate whether the counts (non-zero responses) are related to the linear predictors. The expected value and the variance of the response *y* for a ZIP model are $$E(y_{i})=\mu (1-\theta )$$ and $$Var(y_{i})=\mu (1-\theta )\times (1+\theta \mu ^2)$$ respectively. The model building involves an iterative process which is performed using the MASS package in R. The detail about derivation and application of ZIP model to count responses is available in^48^. To model birth counts, we use four different regression models namely Poisson, NB, ZIP, and ZIP inflation on full data obtained from PDHS-2017-18. We obtain 3 different models corresponding to 3 responses as follows;

- Model 1: Taking 1-year births as responses
- Model 2: Taking 3-years births as responses
- Model 3: Taking 5-years births as responses.

For data with count responses, the regression model utilizes the maximum likelihood (ML) method for estimation of the parameters. Reference^51^ provided a practically understandable introduction of ML estimation. The estimates of all coefficients are obtained by using an iterative set of procedures for estimating parameters. All the ML estimation results are converged and found a unique set of values for each coefficient.

### Birth count regression models under classical approach

After screening different possible determinants of birth from DHS dataset, we select significant variables for fitting final Poisson, NB, ZIP and ZINB models. The estimated coefficients for Models-1, 2, and 3 are presented in Tables 2, 3, and 4 respectively to quantify the impact of different determinants on the number births. The predictor ’region’ is included to obtain the estimates and making comparison of different regions in terms of fertility measures. Other predictors includes the most significant indicators^10^ after excluding the insignificant determinants available in PDHS 2017-18. The standard error for estimated coefficients are reported in parentheses with different codes of significance (Significance codes: 0 ’***’ 0.001 ’**’ 0.01 ’*’ 0.05 ’.’ 0.1 ’ ’ 1). The results provide sufficient evidence that the estimated coefficients for all variables except Sindh, Prof$$\_$$tech, and Prof$$\_$$Agr have a significant effect on the number of 1-year births. The variables Res$$\_$$Age, Residence, and Age$$\_$$husbund show negative estimated coefficients for the Poisson model which supports the argument that the number of births during 1-year period to a woman decreases with the age of mother and is also higher for rural areas. The number of births for urban areas is $$\exp {(-0.1021)}=0.903$$ times the number of births in rural areas assuming no changes in other factors. The likelihood value and its transformations (like AIC and BIC) are used for comparison of the fitting power of competing models^44^. The variable on the region of the respondent is reconstructed into 5 dummies leaving Punjab as the base category. For interpreting the dummies, we follow recommendations given in^52^. The dummy variable corresponding to the province Khyber Pakhtunkhwa (KPK) has negative estimated coefficient $$(-0.0926)$$ with a standard error of 0.035 indicating lower birth exposure in KPK (KPK=1) as compared to Punjab (KPK=0) assuming all other factors as fixed. While the coefficients are $$-0.088$$ and $$-0.05$$ with standard errors 0.021 and 0.015 for Poisson Model 2 and 3 respectively. Similarly, one can distill from Tables 2, 3 and 4 that, after adjusting other variables, the average births during 1, 3 and 5-year period are respectively exp($$-0.0926) = 0.9115$$, exp($$-0.088) = 0.92$$ and exp(−0.05) = 0.96 times of the average births in Punjab. The 95 % confidence intervals for the true effects are $$ (-0.1612, -0.024)$$, $$(-0.12916, -0.04684)$$ and ($$-0.079, -0.021$$) for Poisson Models 1, 2 and 3 respectively. The 95% confidence intervals for the relative rates (exponentiated estimates) are (0.851, 0.976), (0.879, 0.954) and (0.924, 0.98).

The regression coefficient associated with the wealth index (WI) is approximately −0.13 for all models. As WI is dummy coded, the negative sign shows that the average number of births for those who have positive WI is smaller than for those who have negative WI. Similarly, for all three responses, the coefficients for all dummies corresponding to different groups of the contraceptive methods turn negative denoting the number of births for those who use any one of the contraceptive methods is smaller than those who use no contraceptive method. For example, the average births to the women who use contraceptive pills have exp($$-0.358)=0.70$$ times of the average number of births to the women who don’t use any method at all. Further, the ratio of births during 1-year period between those who use male contraceptives and those who use female contraceptives is 0.2383 with exp($$-0.102+0.77885) = 1.97$$ showing that the number of births during 1-year period for those who use male contraceptive method have more birth than those who use a female contraceptive method. The same interpretation for the coefficients corresponding to contraceptive methods can be done with a slight change in the estimated values and standard errors. Further, the average number of 1, 3, and 5-year births to women belonging to an agriculture background are respectively 1.07, 1.06, and 1.123 times higher than those who do not work. However, the regression coefficient for dummy corresponding to pregnancy termination (Preg_term_new) is insignificant. The (1-$$\alpha $$)% confidence interval corresponding to each exponentiated coefficient can be constructed after obtaining confidence interval for the coefficients given in Tables 2.

The method introduced in^52^ for interpreting dummy variables cannot be extended for the interpretation of the coefficients corresponding to the continuous predictors. For the education level variable, the regression coefficient is 0.095. To see one level change in education level, we put $\triangle =1$ into the formula $100 \times [0.095 \times 1-1] = 10$ indicating that there is a $10\%$ ($7.6\%$ for 3-years births and $5.6\%$ for 5-years) increase in the expected number of 1-year births for a unit increase in education level. For the number of children already living in the same household, the regression coefficient is 0.025 with $100\times [exp(0.025\times 1)-1]= 25.3$ showing that there is a $25.3 \%$ increase in the average number of 1-year births with increase of one child in the family. The percentage changes in births during 3-years and 5-years periods before the interview have not been reported to reduce the length of the article. Similarly, the percentage change in the expected births during 1-year period with one unit change in the variable Res_age, Age_husbnd, Num_daughter, MTFBI are 9.7%, 1.64%, 2.5%, 26.905%, and 0.5% respectively. The percentage change in the expected births during 3-years period with one unit change in the variable Res_age, Age_husbnd, Num_daughter, MTFBI are 8.9%, 1.41% 2.14%, 23%, and 0.5% and the percentage change in the expected births during 5-years period with one unit change in the variable Res_age, Age_husbnd, Num_daughter, MTFBI are 7.9%, 1.14%, 1.78% 22.4%, and 0.435084% respectively.

Figure 2 displays the effect of factors on birth counts for the 3 models. The length of bars shows the dispersion in the estimates while the center shows the exponentiated average change in births. The vertical line on the center divides the variables with negative and positive coefficients. The variables with insignificant effects are very close to the vertical line. The bars corresponding to 1-year births for Con_IUD and Con_Female show a highly significant decrement in birth while using these contraceptive methods.

**Table 2:** Estimated coefficients and their standard errors for Model 1.

	Poisson	NB	ZIP-Poisson	ZIP-Inflation
(Intercept)	1.6803 (0.0597***)	1.7026 (0.061***)	0.9648 (0.0678***)	$-$18.4758 (1.253***)
Res_age	$-$0.1021 (0.0027***)	$-$0.1025 ( 0.0027***)	$-$0.0763 (0.003***)	0.4632 (0.029***)
Residence	$-$0.1046 ( 0.0242***)	$-$0.1062 (0.0247***)	$-$0.0609 (0.0255*)	0.8164 (0.23***)
Age_husbnd	$-$0.0165 (0.0021***)	$-$0.0168 (0.0022***)	$-$0.0103 (0.0022***)	0.1371 (0.0167***)
Sindh	0.0037 (0.03086)	0.0029 (0.0315)	$-$0.0408 (0.0329)	$-$0.6159 (0.288*)
KPK	$-$0.0927 ( 0.0352**)	$-$0.0932 ( 0.0358**)	$-$0.0425 (0.0373)	1.2435 ( 0.282***)
Balochistan	$-$0.1234 ( 0.0354***)	$-$0.1223 (0.0361***)	$-$0.2214 (0.036***)	$-$3.9746 ( 0.632***)
ICT	0.1005 ( 0.0482*)	0.1008 ( 0.049*)	0.1339 (0.0537* )	0.3287 (0.3898)
FATA	$-$0.1239 (0.041**)	$-$0.1239 (0.042**)	$-$0.1361 (0.0424**)	$-$0.4029 (0.48062 )
Edu	0.095 (0.0126***)	0.0952 (0.0128***)	0.0852 (0.0134***)	0.0105 (0.1075)
WI	$-$0.1288 (0.0278***)	$-$0.1313 (0.028***)	$-$0.0659 (0.03*)	0.9140 (0.276***)
Num_mem	0.0246 (0.0019***)	0.025 (0.002**)	0.014 (0.0021***)	$-$1.1292 (0.0938***)
Num_daughter	0.2383 (0.0074***)	0.2392 (0.0076***)	0.1892 (0.008***)	0.14621 (0.0815)
MTFBI	0.0049 (0.0005***)	0.0049 (0.0005***)	0.004 (0.0005***)	$-$0.0125 (0.0033*** )
Cont_Pill	$-$0.3583 (0.071***)	$-$0.3617 (0.072***)	$-$0.3603 (0.074***)	1.3378 (0.5268*)
IUD	$-$1.0966 (0.1002***)	$-$1.1004 (0.101***)	$-$1.0272 (0.1133***)	2.1574 (0.7578**)
Cont_Inj	$-$0.3897 (0.0576***)	$-$0.3951 (0.0586***)	$-$0.4149 ( 0.0608***)	0.8401 (0.5885)
Cont_female	$-$0.7789 ( 0.0605***)	$-$0.7789 (0.061***)	$-$0.7821 (0.067***)	0.2787 (0.3572)
Cont_male	$-$0.1017 (0.0356**)	$-$0.105 (0.0364**)	$-$0.1014 (0.0396*)	0.9052 (0.354*)
Cont_withdrawal	$-$0.3429 ( 0.0436***)	$-$0.3454 (0.0443***)	$-$0.2832 (0.048***)	1.7008 (0.3295***)
Cont_other	$-$0.2861 ( 0.076***)	$-$0.2918 (0.0778***)	$-$0.1299 (0.0855)	3.3304 (0.4941***)
Preg_term_new	$-$0.0304 (0.0270)	$-$0.0312 (0.0276)	0.004 (0.0283)	0.4088 (0.2312)
Prof_tech	$-$0.0762 (0.0798)	$-$0.0756 (0.0811)	$-$0.1977 (0.09*)	$-$2.823 (1.0382**)
Prof_Agr	0.0687 (0.0535)	0.0648 (0.0547)	0.112 (0.0587)	0.9415 (0.519)
Prof_other	$-$0.2996 (0.0433***)	$-$0.3031 (0.044***)	$-$0.1456 (0.0474**)	2.2848 (0.3206***)

*, **, and *** refer significantly at 5%, 1% and 0.1% levels respectively.

**Table 3:** Estimated coefficients and their standard errors for Model 2.

	Poisson	NB	ZIP-Poisson	ZIP-Inflation
(Intercept)	2.398 (0.0360***)	2.419 (0.0368***)	1.756 (0.0417***)	$-$17.226 (0.6531***)
Res_age	$-$0.094 (0.0016***)	$-$0.094 (0.0016***)	$-$0.0674 (0.0018***)	0.4514 (0.017***)
Residence	$-$0.0877 (0.014***)	$-$0.0889 (0.015***)	$-$0.0499 (0.015**)	0.658 (0.142***)
Age_husbnd	$-$0.0142 (0.0013***)	$-$0.0143 (0.0013***)	$-$0.0112 (0.0013***)	0.0652 (0.009***)
Sindh	$-$0.0039 (0.0185)	$-$0.0034 (0.0189)	$-$0.0352 (0.0196*)	$-$0.3242 (0.1608*)
KPK	$-$0.0879 (0.021***)	$-$0.0886 (0.0214***)	$-$0.0804 (0.022***)	0.336 (0.1834)
Balochistan	$-$0.101 (0.0214***)	$-$0.0985 (0.0218***)	$-$0.1754 (0.0224***)	$-$1.7996 (0.3302***)
ICT	0.0519 (0.029.)	0.0517 ( 0.03.)	0.0395 (0.031)	$-$0.3946 (0.205.)
FATA	$-$0.0649 (0.0246**)	$-$0.064 (0.0251*)	$-$0.0646 (0.0255*)	0.2322 (0.2480)
Edu	0.0731 ( 0.0076***)	0.0737 (0.0077***)	0.0547 (0.0079***)	$-$0.1968 (0.0627**)
WI	$-$0.1249 (0.0166***)	$-$0.1279 (0.017***)	$-$0.0374 (0.018*)	1.5051 ( 0.155***)
Num_mem	0.0211 ( 0.0012***)	0.0215 (0.0012***)	0.0127 (0.0013***)	$-$0.7039 (0.042***)
Num_daughter	0.207 ( 0.005***)	0.2079 ( 0.005***)	0.1567 ( 0.005***)	$-$0.1655 ( 0.0504**)
MTFBI	0.0046 ( 0.0003***)	0.0046 (0.0003***)	0.0033 (0.0003***)	$-$0.0166 (0.0023***)
Cont_Pill	$-$0.0417 (0.0379)	$-$0.0419 (0.0386)	$-$0.0580 (0.0399)	1.0315 (0.295***)
IUD	$-$0.2801 (0.042***)	$-$0.2819 (0.043***)	$-$0.3399 (0.045***)	$-$0.263 (0.429)
Cont_Inj	0.0624 (0.029*)	0.0635 (0.03*)	$-$0.0163 ( 0.0304)	$-$1.7268 (0.62**)
Cont_female	$-$0.5299 (0.033***)	$-$0.529 (0.033***)	$-$0.457 (0.038***)	0.8415 (0.187***)
Cont_Male	0.037 (0.021.)	0.0368 (0.0216.)	0.0103 (0.0225)	0.3884 (0.188*)
Cont_withdrawal	$-$0.1376 ( 0.025*)	$-$0.138 (0.025***)	$-$0.176 (0.026***)	0.0787 (0.1945)
Cont_other	0.0465 (0.0411)	0.0459 (0.0419)	0.0623 (0.0438)	1.1738 (0.28***)
Preg_term_new	$-$0.0485 (0.016**)	$-$0.0489 (0.016**)	$-$0.0193 (0.0169)	0.4537 (0.15**)
Prof_tech	0.0923 ( 0.044*)	0.0962 (0.045*)	0.0179 (0.047***)	$-$1.5429 (0.336*** )
Prof_Agr	0.0596 (0.032.)	0.0587 (0.033.)	0.1091 (0.035**)	1.0635 (0.263***)
Prof_Other	$-$0.1290 (0.024***)	$-$0.1294 (0.025***)	$-$0.0838 (0.03**)	0.6009 (0.184**)

*, **, and *** refer significantly at 5%, 1% and 0.1% levels respectively.

**Table 4:** Estimated coefficients and their standard errors for Model 3.

	Poisson	NB	ZIP-Poisson	ZIP-Inflation
(Intercept)	2.5448 (0.027***)	2.5637 (0.027***)	2.00 (0.03***)	$-$16.395 (0.48***)
Res_age	$-$0.0822 (0.001***)	$-$0.0828 ( 0.001***)	$-$0.0585 (0.0013***)	0.4309 (0.0129***)
Residence	$-$0.0947 (0.011***)	$-$0.0959 (0.011***)	$-$0.0505 (0.011***)	0.9489 (0.105***)
Age_husbnd	$-$0.0114 (0.0001***)	$-$0.0115 (0.0001***)	$-$0.0089 (0.0001***)	0.0607 (0.007***)
Sindh	$-$0.0035 (0.0138)	$-$0.0031 (0.014)	$-$0.0294 (0.0145*)	$-$0.1791 (0.1161)
KPK	$-$0.0502 (0.016**)	$-$0.0505 (0.016**)	$-$0.0598 (0.016***)	$-$0.0199 (0.1294)
Balochistan	$-$0.045 (0.016**)	$-$0.0427 (0.0161**)	$-$0.113 (0.0164***)	$-$1.2969 (0.207***)
ICT	0.0293 (0.0219)	0.0294 (0.0222)	0.0319 (0.0233)	$-$0.0638 (0.1503)
FATA	$-$0.0579 (0.018**)	$-$0.0565 (0.019**)	$-$0.0864 (0.019***)	$-$1.0513 (0.264***)
Edu	0.0542 (0.0057***)	0.0548 (0.0058***)	0.0345 ( 0.006***)	$-$0.3233 (0.049***)
WI	$-$0.1343 (0.012***)	$-$0.1368 (0.013***)	$-$0.0707 (0.013***)	1.0432 (0.11***)
Num_mem	0.0176 (0.001***)	0.0179 (0.001***)	0.0105 (0.001***)	$-$0.6432 (0.034***)
Num_daughter	0.2022 (0.003***)	0.2032 (0.003***)	0.1525 (0.004***)	$-$0.2735 (0.037***)
MTFBI	0.0043 (0.0002***)	0.0044 (0.0002***)	0.0030 (0.0002***)	$-$0.0182 (0.0016***)
Cont_Pill	$-$0.0079 (0.0281)	$-$0.009 (0.0286)	$-$0.0206 (0.0293)	1.2046 (0.222***)
IUD	$-$0.0401 (0.028)	$-$0.0417 (0.029)	$-$0.1076 ( 0.03***)	$-$0.5326 (0.285.)
Cont_Inj	0.1331 (0.021***)	0.1346 (0.022***)	0.0423 ( 0.0216.)	$-$4.6319 (0.84***)
Cont_female	$-$0.2751 ( 0.0215***)	$-$0.2747 (0.022***)	$-$0.1903 (0.024***)	0.9573 (0.121***)
Cont_male	0.0401 (0.016*)	0.0409 ( 0.0164*)	$-$0.009 (0.0168)	$-$0.1549 (0.1532)
Cont_withdrawal	$-$0.0075 (0.0176)	$-$0.0066 (0.0179)	$-$0.085 (0.019***)	$-$0.89 (0.17***)
Cont_other	0.0971 (0.03**)	0.0979 (0.031**)	0.0746 (0.032*)	0.538 (0.222*)
Preg_term_new	$-$0.0409 (0.012***)	$-$0.0416 (0.0124***)	$-$0.0265 (0.012*)	0.1889 (0.108.)
Prof_tech	0.0545 (0.0334)	0.057 (0.034.)	$-$0.0239 (0.035)	$-$1.6005 (0.294***)
Prof_Agr	0.1163 (0.023***)	0.116 ((0.024***))	0.1614 (0.025***)	0.973 (0.179***)
Prof_Other	$-$0.0771 (0.017***)	$-$0.0772 (0.018***)	$-$0.0381 (0.0185*)	0.4939 (0.127***)

*, **, and *** refer significantly at 5%, 1% and 0.1% levels respectively.

A comparison of Poisson, NB, and ZIP are provided in Table 5 for three different responses. The minimum, maximum and mean residuals for each model are reported. The minimum residual is observed at extreme (i.e. -1.0731, -1.7707, and -2.6435) for the ZIP model. While maximum residual is observed at extreme (i.e. 51.793, 15.0487, and 9.5761) for NB. Further to see the model performance -2logliklihood and AIC values are also reported. The AIC values are observed smaller for ZIP as compared to other models considered in this study (Table 5).

**Table 5:** Residual Comparison of Models.

	Poiss	NB	ZIP
Model 1			
Minimum	$-$1	$-$1	$-$1.0731
Mean	$-$0.122	$-$0.121	0.0000
Maximum	51.125	51.793	2.5155
2logLikelihood	$-$42984.63	$-$43307.25	$-$42011.64
AIC	43035	43359	42036.64
Model 2			
Minimum	$-$1	$-$1	$-$1.7707
Mean	$-$0.0978	$-$0.0969	0.0001
Maximum	14.8876	15.0487	3.0787
2logLikelihood	$-$77067.93	$-$77564.3600	$-$75013.42
AIC	77118	77616	75038.42
Model 3			
Min.	$-$1	$-$1	$-$2.6435
Mean	$-$0.0754	$-$0.0747	0.0001
Max.	9.4748	9.5761	4.1984
2logLikelihood	$-$106392.9	$-$106877.2	$-$102943.6
AIC	106443	106929	102964.4016

**Figure 3:**
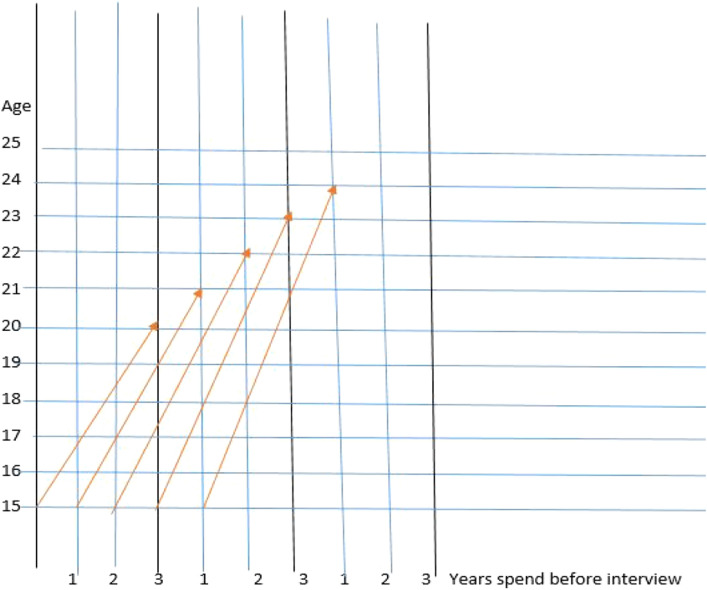
Illustration of birth history data on a Lexis diagram (each birth can fall in one of the given categories then person-years are calculated according to these categories) is given in Fig. 3. Years spent by respondents before interview are shown on X-axis in 3-year periods. While age of respondents are shown on Y-axis the first arrow begins from 15 years age and end at 20 years inferring that birth falling in this category, i.e. area between first and second arrows before the first vertical line corresponding to 3, are counted as exposure for group 15-19.

### Birth count regression models under bayesian framework

In this section, we analyze the birth history data using the PRM, previously run under frequentist’s point of view in a Bayesian framework by adding a normal prior on the coefficients of the linear log-mean function as given in (6) i.e. $\beta _{j}\sim N(0,1\times 10^{-3})$ for all $j=0,1,2,...,24$. The analysis is done in R-library (rjags) Just Another Gibbs Sampler (JAGS) taking three chains. We initialize the model and run the burn-in period and the model is updated 100 times and the number of iterations is taken 1,000. We take relatively fewer iterations as the number of nodes is 50,495 which leads to a longer time in running the complete model. After deciding the burn-in period, we simulate the samples that will keep. We re-run the model for checking the convergence and to see the auto-correlation. The deviance information criterion is also obtained for each model.

**Table 6:** Posterior means, standard deviation and effective size for regression coefficients.

Variables	Model 1			Model 2			Model 3		
	Mean	STD	Eff Size	Mean	STD	Eff Size	Mean	STD	Eff Size
(Intercept)	1.6775	0.0611	38.7081	1.677	50.5251	0.0611	2.5457	0.024	51.1951
Res_age	$-$0.1017	0.0021	36.3485	$-$0.1017	24.1892	0.0021	$-$0.0819	0.0008	41.7633
Residence	$-$0.1048	0.0228	1534.81	$-$0.1048	571.98	0.0228	$-$0.0942	0.0103	639.54
Age_husbnd	$-$0.0168	0.0016	32.75	$-$0.0168	27.51	0.0016	$-$0.0116	0.0006	53.72
Sindh	0.0037	0.0301	696.8	0.0037	805.95	0.0301	$-$0.0037	0.0138	634.94
KPK	$-$0.0934	0.0355	488.83	$-$0.0934	501.22	0.0355	$-$0.0508	0.0159	437.95
Balochistan	$-$0.1249	0.0358	653.91	$-$0.1249	615.68	0.0358	$-$0.0453	0.0161	518.26
ICT	0.0998	0.0476	1390.18	0.0998	1074.85	0.0476	0.029	0.0217	1318.52
FATA	$-$0.1242	0.0409	708.39	$-$0.1242	792.49	0.0409	$-$0.0582	0.0186	702.03
Edu	0.0942	0.0126	485.61	0.0942	529.95	0.0126	0.0544	0.0058	486.34
WI	$-$0.129	0.0266	470.94	$-$0.129	449.55	0.0266	$-$0.1351	0.0122	512
Num_mem	0.0248	0.002	279.92	0.0248	318.58	0.002	0.0176	0.0009	232.89
Num_daughter	0.2379	0.0081	288.75	0.2379	318.44	0.0081	0.2021	0.0032	333.04
Mar_first	0.0049	0.0005	639.47	0.0049	464.98	0.0005	0.0043	0.0002	559.27
Cont_Pill	$-$0.3625	0.0715	1505.22	$-$0.3625	1603.4	0.0715	$-$0.0081	0.0286	1524.9
IUD	$-$1.1026	0.1011	1855.87	$-$1.1026	1382.57	0.1011	$-$0.041	0.0278	1611.72
Cont_Inj	$-$0.3891	0.0581	1673.25	$-$0.3891	1374.7	0.0581	0.133	0.0215	1926.15
Cont_female	$-$0.7811	0.0619	1465.15	$-$0.7811	1665.05	0.0619	$-$0.2768	0.0219	1393.43
Cont_male	$-$0.104	0.0353	1280.29	$-$0.104	1327.15	0.0353	0.0409	0.016	1231.12
Cont_with\_draw	$-$0.3441	0.0437	1499.29	$-$0.3441	1252.7	0.0437	$-$0.0081	0.0178	1417.29
Cont_other	$-$0.2888	0.0767	685.28	$-$0.2888	1626.67	0.0767	0.0964	0.0309	1388.1
Preg_term	$-$0.0304	0.0281	248.15	$-$0.0304	278.01	0.0281	$-$0.0412	0.0127	228.99
Prof_tech	$-$0.0785	0.0785	1641.41	$-$0.0785	1560.07	0.0785	0.0538	0.0331	1374.54
Prof_Agr	0.0689	0.0537	1394.11	0.0689	1204.17	0.0537	0.1156	0.0232	1375.04
Prof_other	$-$0.3014	0.0422	1585.41	$-$0.3014	1434.15	0.0422	$-$0.0773	0.0172	1398.44

**Table 7:** Model Comparison.

	1-years	3-years	5-years
RES-mean	0.00061	0.33003	0.00107
Res-SD	0.13137	0.34963	0.58909
Mean deviance	43015	77117	106448
Penalty	24.08	25.26	25.56
Penalized deviance	43039	77142	106474

The 2nd, 5th and 8th columns of Table 6 give the posterior means of each coefficient, 3rd, 6th and 9th columns provide their standard deviation (standard errors), and 4th, 7th and 10th columns give effective sample sizes. One can notice that the posterior means almost match with the estimated coefficients corresponding to each variable with a slight reduction in standard error. The standard error for the “Residence” variable with Poisson model with 1-year period births as the response is 0.0242 while the corresponding posterior standard deviation for the same coefficient under MCMC is 0.0228. After running MCMC, we need to confirm whether MCMC sampler covers the parameter space efficiently, i.e. it does not accept or reject too many proposals. A detail discussion of MCMC diagnostics can be found in^53,54^ and^55^ etc. If the MCMC rejects too many proposals, we need a large number of simulations to generate a considerable number of parameter samples. On the other end, if a large number of proposals are accepted, we cannot find much information about the parent distribution. Trace plots are the important tools for assessing the mixing of a chain. Figures 9, 10, 11, 12, 13, 14, 15 show the trace plots corresponding to each coefficient which provide evidence of the presence of randomness (lack of pattern) in data. The trace plots corresponding to intercept $\beta _{0}$, and two coefficients $\beta _{1}$ and $\beta _{3}$ reflect a slight lack of randomness while the trace plots corresponding to all other coefficients provide enough evidence of randomness. We provide the trace plot corresponding to the model for the births during 1-year period. Density plots are smoothed histograms of the samples, i.e. they show the function that we are trying to explore. Observing Figures 9, 10, 11, 12, 13, 14,15 one can also see the behavior of posterior densities for each coefficient through density plots. An alternative way to check for convergence of the estimates is to look at the auto-correlations among the samples obtained from MCMC. The lag-*l* auto-correlation is the correlation between every sample and the sample *l* steps earlier (which become smaller as *l* increases) i.e. considering samples as independent. On the other hand, if this auto-correlation remains constant (high) for higher values of *l* too, then the situation depicts a higher correlation between every sample and the sample *l* steps before. The autocorrelation plots corresponding to each coefficient for the three models are obtained from MCMC sampling. The plots for the model with 1-year period births as response variable are reported in Appendix.

**Figure 4:**
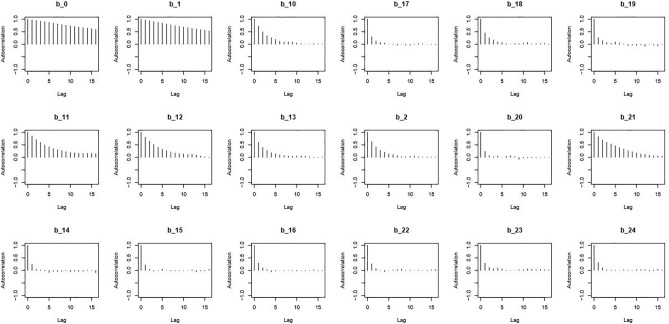
Auto Correlation of coefficients Plot 1.

**Figure 5:**
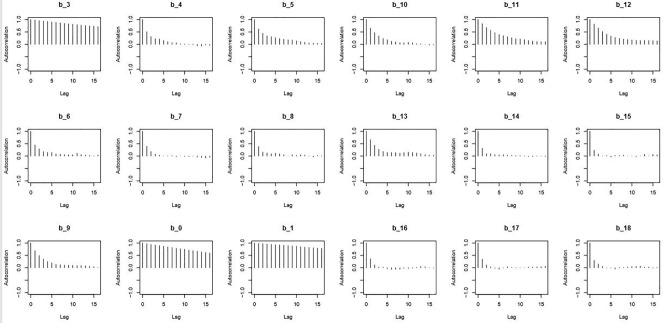
Auto Correlation of coefficients Plot 2.

**Figure 6:**
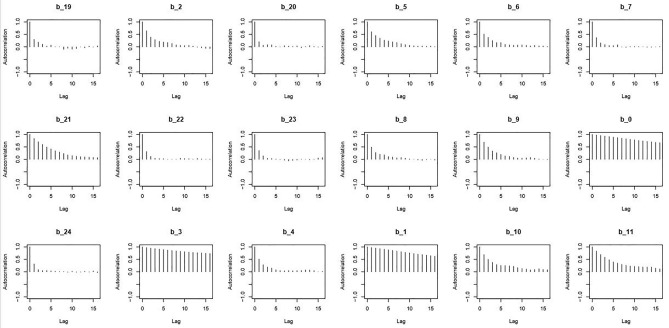
Auto Correlation of coefficients Plot 3.

**Figure 7:**
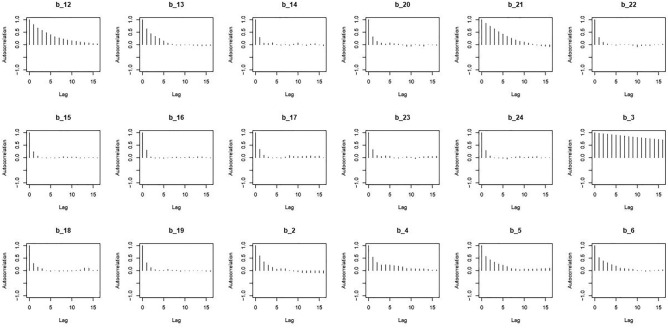
Auto Correlation of coefficients Plot 4.

**Figure 8:**
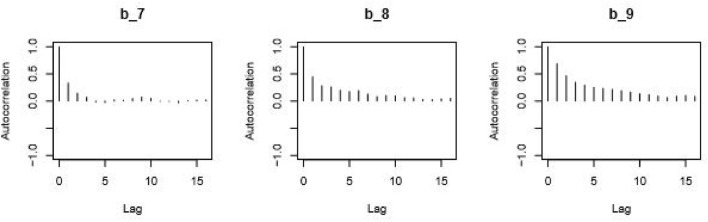
Auto Correlation of coefficients Plot 5.

**Figure 9:**
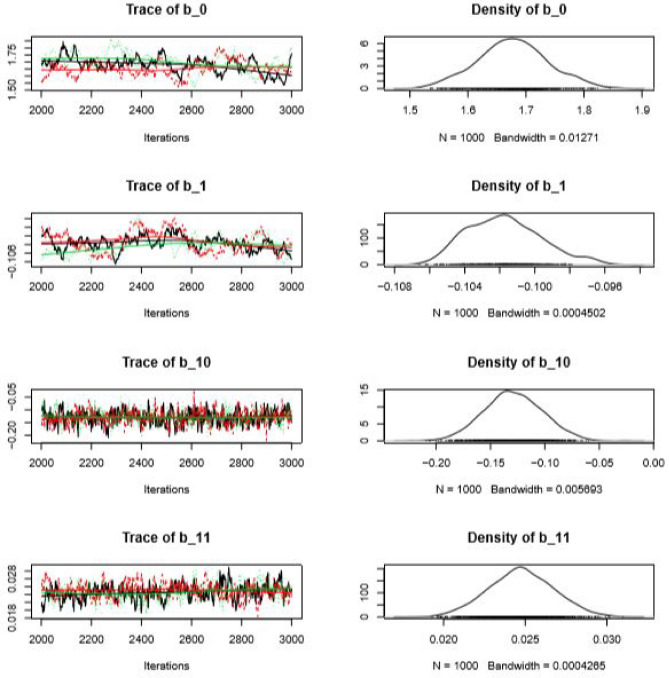
Trace Plot 1-A.

**Figure 10:**
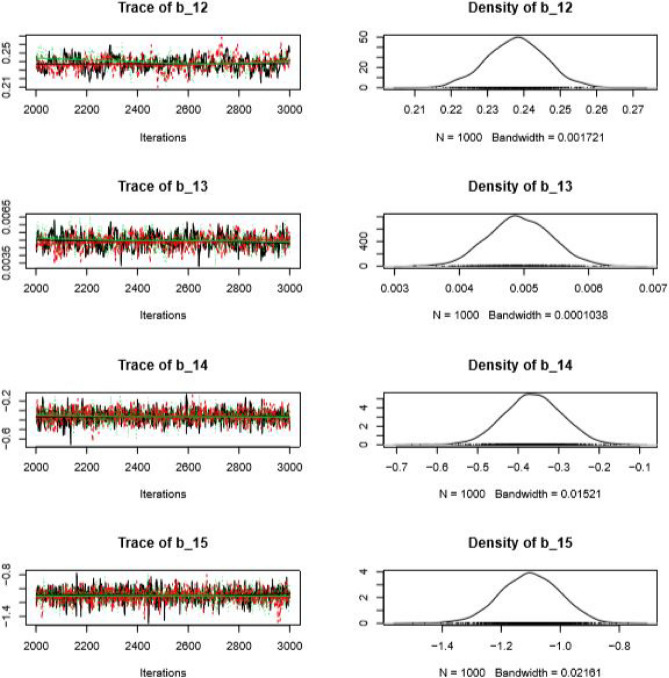
Trace Plot 1-B.

**Figure 11:**
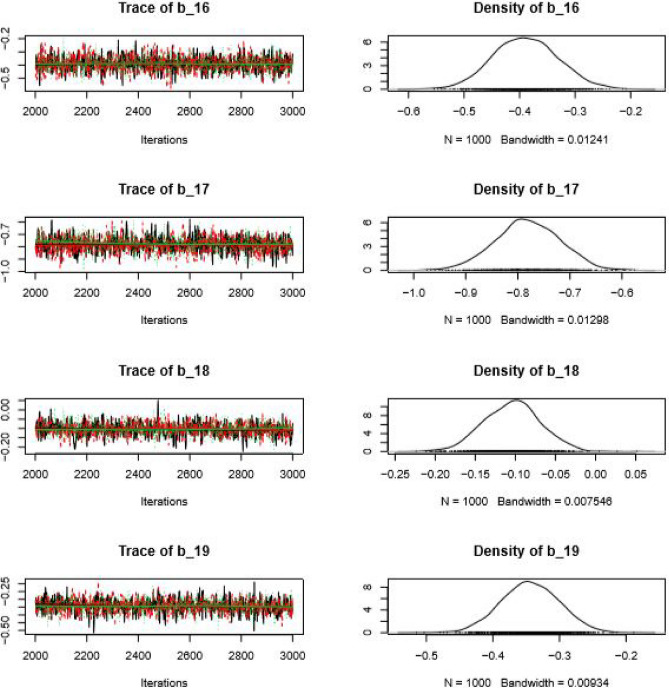
Trace Plot 1-C.

**Figure 12:**
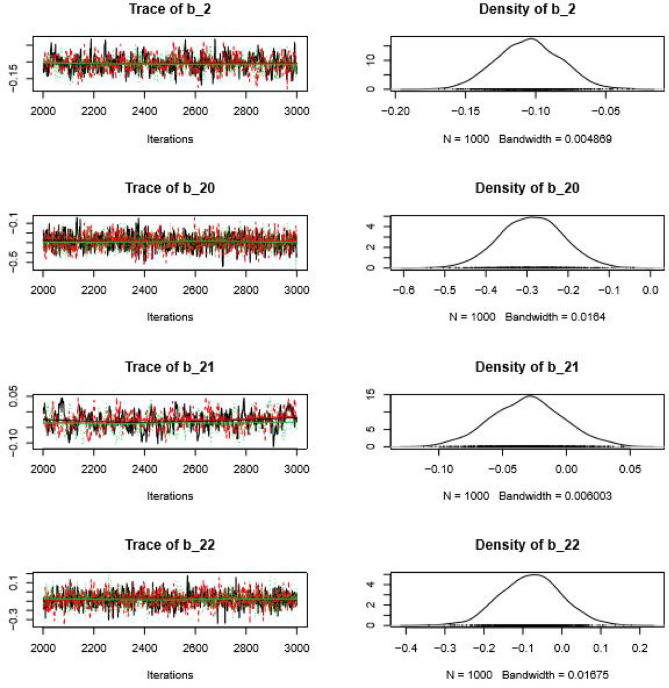
Trace Plot 1-D.

**Figure 13:**
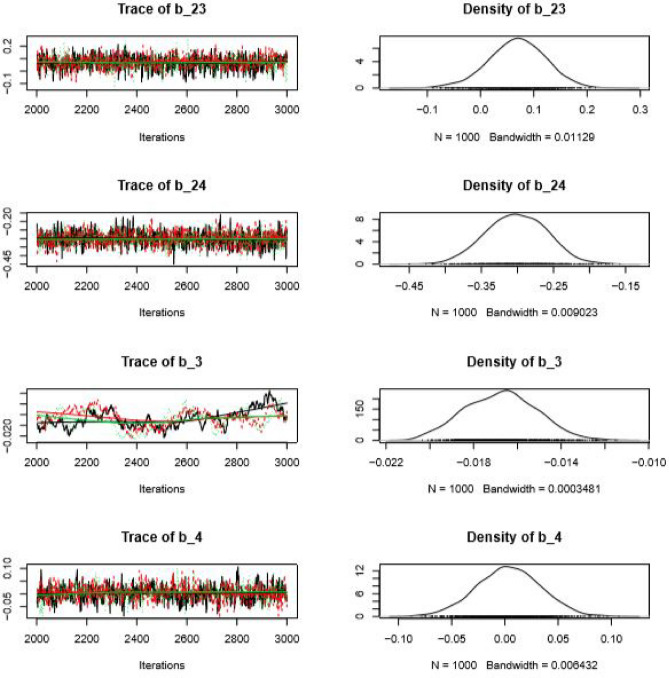
Trace Plot 1-E.

**Figure 14:**
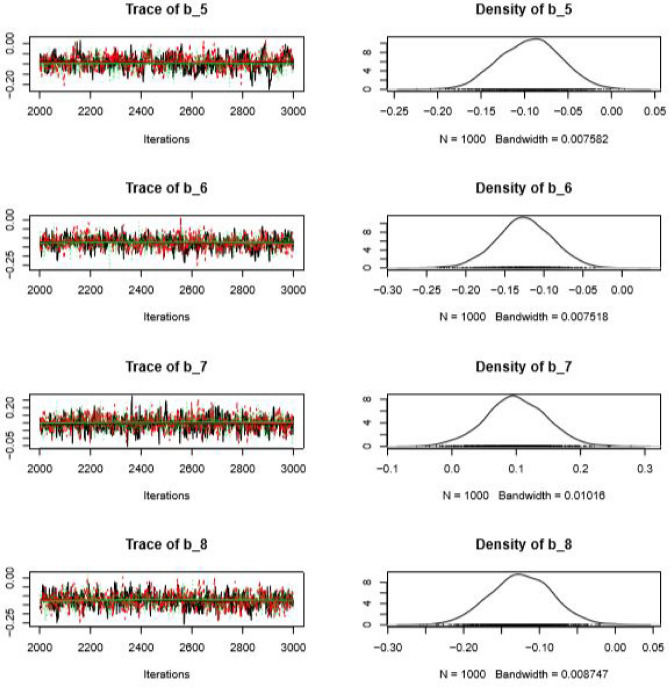
Trace Plot 6.

**Figure 15:**
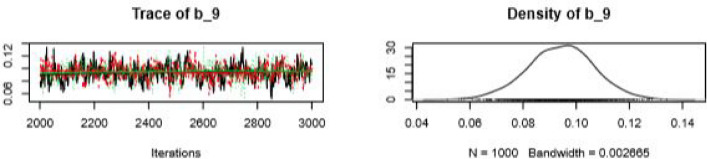
Trace Plot 7.

The autocorrelation plots are displayed in Figs. 4, 5, 6, 7, 8 (see Appendix). It can be noticed that the auto-correlation goes down for all coefficients with increase in *l*. However, the auto-correlation plots corresponding to the intercept and the coefficient of age indicate the presence of auto-correlation. This auto-correlation can be reduced by thinning the MCMC chains, i.e. by discarding *n* samples for every sample that we keep. The thinning of the MCMC chain is not of much use unless we want to reduce the memory and storage space in long chains. With this argument, one should keep only one out of ten samples instead of thinning the chain because this is more efficient, concerning the Effective Sample Size (ESS), to run only one chain 10 times as long, it will take 10 times more storage space. A more reliable estimate for burn-in cut-off is through the ESS. An ESS is the number of independent samples with the same estimation power as the number of autocorrelated samples. The burn-in samples are the samples that have not much information, and if the period of burn-in is estimated to be short enough then this will lead to a reduction in the ESS. On contrary, if the period of burn-in is estimated to be much longer, again causes in reduction of the ESS as informative samples are being isolated. An increase in ESS should be with the optimal estimate of the burn-in are highly recommended in practical estimation procedures. The burn-in samples can be assessed from the trace plots and ESS. Table 6 shows that the ESS all coefficients, except $\beta _{0}$, $\beta _{1}$ and $\beta _{3}$ which are 38.7, 36.3, and 32.75 respectively, are large enough to continue. The ESS for the coefficient of Cont_Inj is maximum with 1,855. Finally, we see the predictive power of our models by checking at deviances. The most widely used tool for checking the predictive power of a model is the Deviance Information Criterion (DIC). It is an estimate of the expected predictive error of the model. Table 7 gives the mean deviances for the three models. The DIC value is least (i.e. 43,015) for the model with the number of birth during 1-year period as the response. The penalized deviance for the same model with the penalty of 24.08 is 43,039 which is slightly larger than the deviance without penalty.

## Model-based estimation of fertility indicators

In PDHS final report^43^, ASFRs are obtained according to the formula given in Equations (1) and (2) for each age group. The mother’s age is computed, in century month code (CMC) format, by taking the difference of the date of the interview and the mother’s date of birth. Births are then tabulated by age group after converting the ages into years. Similarly, the denominator in ASFR is women-years of exposure in the five-year age group during the 3-years time-period. A woman can expose to several age groups in the given period, with varying lengths of the period. For the 3-years period, a woman will contribute to at most two five-year age groups during the 36-months period. For further details related to the allocation of women to the extreme age groups (higher and lower), readers are referred to^43^.

We developed R codes for estimating ASFR under a model-based approach after converting the individual (IR) data to person-year data. For tabulation of person-years, each woman is tallied twice, once in the lower age group aggregating lower age group exposure and once according to the higher age group summing the exposure she contributes to the higher age group. In computing fertility rates, we use only ever-married samples without taking the “all-women factor” under a model-based approach. Hence the interpretation of the rates is done based on birth per ever-married woman only. The total exposure in each age group is the sum of the exposure in each age group tallying from the first and second. After obtaining ASFR, it is straightforward to obtain TFR, GFR, and GRR using formulae given in Equations (2), (3) and (4). We obtained fertility measures i.e. ASFR, TFR, GFR, and GRR using predicted responses obtained from the regression models after partitioning data into sampled and non-sampled parts. A bootstrap sampling procedure was used to study the design-based properties of the estimated fertility rates PDHS 2017-18 women re-coded data. The calculations are documented in the following algorithm.

**Algorithm 1:**
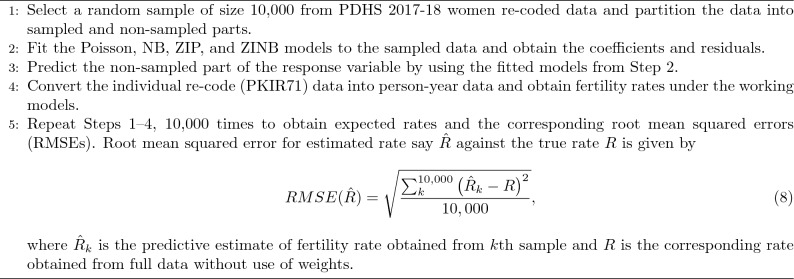
Boot-strapped algorithm for birth rates estimation

**Table 8:** Model-Based ASFR for Ever-married Women in Pakistan.

AGE	Poisson	NB	ZIP	ZINB	FULL DATA
	PAKISTAN				
15–19	222.72 (4.89)	223.79 (7.09)	**203.66 (3.18)**	$\underline{202.60\,(3.66)}$	178.69
20–24	$\underline{275.08(2.78)}$	266.08 (4.17)	258.54 (2.27)	**258.75 (2.15)**	271.77
25–29	244.48 (2.53)	244.81 (3.42)	$\underline{{\textbf {250.23}}\,{\textbf {(2.04)}}}$	247.94 (2.16)	254.29
30–34	191.69 (2.17)	191.79 (2.88)	**204.78 (1.46)**	$\underline{203.16\,(2.27)}$	202.24
35–39	$\underline{114.42\,(2.54)}$	114.36 (2.91)	**131.72 (2.22)**	130.76 (1.28)	115.66
40-44	54.62 (2.18)	$\underline{54.41\,(3.15)}$	**72.15 (2.98)**	71.49 (1.8)	46.49
45-49	**23.71 (1.18)**	$\underline{23.46\,(1.26)}$	39.60 (2.69)	37.67 (1.94)	14.72

Significant values are bold, underline, boldunderline.

Model-based ASFRs for Pakistan at national and sub-national levels for an ever-married sample along with their RMSE are presented in Table 8, and Tables 9-10 respectively under four different working models. The expected ASFR with the smallest RMSE among four (obtained under four alternative models) is bolded corresponding to each age group for the sub-national and national levels. In majority of the cases, the ZIP model gives relatively smaller RMSE at both national and sub-national levels. Comparing the estimated ASFR with the ASFR obtained from full data in the last column of Tables 8-10, we can notice that the ASFR for age groups 1, 2, 6, and 7 are upward biased for all models i.e. they are estimated larger than the ASFER obtained from full data. The discrepancies in results occurred due to the use of person time data for estimating ASFRs. As for smaller and elder ages we are not able to exactly find the exposure to births and denominator in ASFR might be counted below the actual exposure time. Which can be considered as major limitation of this approach. The expected ASFR with the smallest difference (bias) with the ASFR obtained from full data among four is underlined corresponding to each age group at both national and sub-national levels. For example, the closest estimate for age group 1 is obtained under NB model in Punjab province. From ASFR based on full PDHS data, one can observe that for age group 15-19 the highest average number of births is observed for FATA and the lowest for KPK. Similarly, ASFR can be compared across regions based on full data.

The TFR, GFR, and GRR for ever-married women are provided at sub-national and national levels in Tables 11, 12 and 13 respectively. The TFR for the ever-married sample is observed highest in ICT with 6.26 and lowest in Punjab with 5.44 per 1000 ever-married women. While the TFR for all women given in PDHS 2017-18 report is observed highest in FATA. The TFR obtained for all women using the DHS.rates package are given in Appendix. The estimates obtained under NB, ZIP, and ZINB are more accurate than the ones observed for Poisson which can be noticed from RMSE in Table 11 corresponding to each region. The smallest RMSE is observed at the national level when ZIP is used to model births i.e. 0.0617. Similarly, the predictive estimate for GFR at the sub-national and national level is observed highest for ever-married women in KPK which is 174.95.6 (NB as it is more precise than the other three estimates) births per 1000 married women. The lowest GFR is observed for Punjab with 162 children per 1000 ever-married women. The RMSE is observed highest when the Poisson regression model is used for modeling births in KPK. While for other regions continuing with Poisson, ZIP and NB give almost similar RMSE for estimating GFR at sub-national and national levels. The GFR for full data without model fitting is obtained in the last column of Table 12. The GRR is computed using the proportion of female births (PF) from all age groups using the sex ratio of male to female from full data (PF from census or administrative records can be used for obtaining sex ratio). The GRR is observed higher in Balochistan, Punjab, and KPK as compared to other regions replacement of 2 or more daughters per woman before the death of their mother. RMSE is smaller for Punjab, Sindh, and KPK when the NB model is used for prediction. While it is smaller for the remaining regions when the ZIP model is employed.

**Table 9:** Model-Based ASFR for Ever-married Women by Region.

Age group	Poisson	NB	ZIP	ZINB	FULL DATA
Punjab					
15–19	211.30 (10.14)	$\underline{212.08\,(9.92)}$	**189.93 (6.88)**	193.16 (10.19)	249.19
20–24	269.36 (5.06)	269.81 (6.67)	$\underline{{\textbf {263.32}}\,{\textbf {(5.36)}}}$	263.92 (4.71)	231.78
25–29	$\underline{250.47\,(5.47)}$	250.50 (5.18)	**254.32 (5.15)**	257.03 (5.16)	198.05
30–34	192.09 (4.27)	$\underline{191.97\,(6.68)}$	204.79 (5.54)	**206.77 (4.32)**	186.52
35–39	101.89 (6.08)	101.67 (4.77)	**118.71 (3.54)**	$\underline{120.37\,(5.67)}$	127.90
40–44	44.75 (5.65)	**44.45 (3.14)**	$\underline{63.58\,(4.66)}$	63.18 (3.02)	76.09
45–49	18.96 (2.19)	**18.63 (1.86)**	34.14 (3.05)	$\underline{35.46\,(3.75)}$	39.30
Sindh					
15–19	$\underline{229.492\,(19.53)}$	230.71 (13.78)	212.45 (9.96)	**210.08 (7.74)**	228.2823
20–24	$\underline{268.92(11.21)}$	260.09(5.19)	**250.91 (3.05)**	253.51 (5.94)	265.92
25–29	$\underline{239.79\,(15.25)}$	240.34 (5.84)	**246.13 (4.93)**	245.51 (6.49)	230.04
30–34	$\underline{187.74\,(4.75)}$	187.99 (4.77)	204.97 (6.75)	**200.48 (4.97)**	189.73
35–39	$\underline{109.71\,(8.39)}$	109.55 (7.61)	**130.35 (4.57)**	126.93 (6.22)	111.77
40–44	$\underline{56.39\,(9.19)}$	**56.23 (4.46)**	75.85 (5.16)	72.91 (6.11)	61.27
45–49	$\underline{24.69\,( 3.75)}$	**24.49 (2.20)**	40.31 (8.58)	40.25 (3.43)	27.86
KPK					
15–19	196.37 (7.47)	$\underline{196.96\,(8.96)}$	182.88 (6.65)	**180.38 (5.83)**	207.41
20–24	273.84(5.65)	$\underline{{\textbf {274.39(5.32)}}}$	266.2204 (7.86)	266.69 (9.91)	270.69
25–29	$\underline{263.24\,(5.66)}$	263.44 (8.45)	**267.26 (3.54)**	269.03 (9.70)	246.06
30–34	186.11 (5.45)	$\underline{186.21\,(6.29)}$	201.37 ( 5.12)	**199.68 (4.89)**	191.65
35–39	115.26 (4.49)	$\underline{115.18\,(4.96)}$	**133.56 (4.14)**	132.05 (4.26)	101.75
40–44	45.62 (5.69)	$\underline{{\textbf {45.37}}\,{\textbf {(3.09)}}}$	62.71 (4.37)	63.40 (4.15)	44.12
45–49	22.09 (3.64)	$\underline{21.76 (5.86)}$	36.72 ( 7.69)	**37.93 (3.54)**	19.65
Balochistan					
15–19	250.97 (9.93)	$\underline{252.77\,(10.21)}$	**227.32 (6.09)**	228.34 ( 8.99)	252.46
20–24	$\underline{239.49\,(9.93)}$	240.69 (6.77)	**228.41 ( 7.53)**	229.57 (13.6)	248.72
25–29	213.59 (16.66)	$\underline{214.64\,(9.26)}$	**212.75 (5.94)**	214.75 (7.43)	225.05
30–34	$\underline{188.63\,(6.93)}$	189.42 (10.75)	**194.92 (5.47)**	195.98 (7.55)	188.51
35–39	$\underline{{\textbf {126.13}}\,{\textbf {(5.81)}}}$	126.41 (13.63)	138.17 (6.14)	138.62 (6.56)	119.97
40–44	$\underline{82.22\,(8.32)}$	**82.30 (5.63)**	97.36 (8.54)	95.87 (5.64)	76.17
45–49	$\underline{40.86\,(5.33)}$	**40.74 (5.11)**	54.45 (5.71)	54.74 (5.19)	44.12

Significant values are bold, underline, boldunderline.

**Table 10:** Model-Based ASFR for Ever-married Women by Region (Continued).

AGE	Poisson	NB	ZIP	ZINB	Full Data
ICT					
15–19	266.46 (18.83)	267.62 (18.52)	$\underline{{\textbf {238.3424}}\,{\textbf {(4.26)}}}$	240.76 (15.35)	211.95
20–24	273.47 (9.65)	265.55 (7.62)	$\underline{{\textbf {254.13}}\,{\textbf {(7.09)}}}$	253.14 (13.65)	258.93
25–29	257.72 (7.61)	258.43 (7.09)	**262.23 ( 4.97)**	$\underline{256.34\,(7.09)}$	225.43
30–34	236.78 (8.91)	237.56 (10.36)	**241.53 (7.52)**	$\underline{243.59\,(8.84)}$	186.53
35–39	$\underline{128.49 (14.14)}$	128.59 (6.61)	**139.65 (4.07)**	144.39 (6.77)	102.27
40–44	66.63 (7.54)	$\underline{{\textbf {66.56}}\,{\textbf {(6.67)}}}$	84.66 (9.9)	82.04 (7.55)	48.07
45–49	22.50 (7.33)	$\underline{{\textbf {22.32}}\,{\textbf {(3.89)}}}$	36.01 (9.51)	34.69 (3.68)	21.6777
FATA					
15–19	231.64 (17.46)	$\underline{232.23\,(21.74)}$	**218.18 (7.93)**	215.88 (13.83)	257.99
20–24	289.01 (9.02)	282.27 (8.34)	**285.68 (5.53)**	$\underline{278.52\,(7.99)}$	278.99
25–29	243.32 (13.18)	243.06 (9.76)	**258.94 ( 4.93)**	$\underline{251.47\,(12.35)}$	254.72
30–34	$\underline{187.99 (8.11)}$	187.65 (9.27)	**202.96 (0.926)**	199.85 (6.30)	239.74
35–39	87.37 (5.21)	87.04 (5.88)	**104.09 (1.80)**	$\underline{106.04\,(8.53)}$	141.08
40–44	48.78 (8.00)	48.37 (4.55)	**66.28(1.7)**	$\underline{66.92(8.3)}$	71.13
45–49	18.20 ( 4.60)	$\underline{{\textbf {17.80}}\,{\textbf {(3.39)}}}$	33.85 (4.62)	35.27 (7.89)	16.14

Significant values are bold, underline, boldunderline.

**Table 11:** Model-Based TFR at Regional Level.

REGION	Poisson	NB	ZIP	ZINB	FULL DATA
Punjab	$\underline{5.44 \,(1.01)}$	**5.45 (0.11)**	5.64 (0.516)	5.69 (0.57)	4.43
Sindh	5.58 (1.15)	$\underline{{\textbf {5.55}}\,{\textbf {(0.13)}}}$	5.80 ( 0.45)	5.7484 ( 0.40)	4.432
KPK	$\underline{5.51\, (1.08)}$	**5.52 (0.087)**	5.75 (0.44)	5.75 ( 0.43)	4.432
Bolachistan	$\underline{5.71\,(1.28)}$	5.735 (0.229)	**5.767 (0.228)**	5.78 (0.255)	4.43
ICT	6.26 (1.83)	$\underline{6.23\,(0.64)}$	**6.28 (0.42)**	6.2749 (0.41)	4.432
FATA	5.53 (1.11)	$\underline{5.49\,(0.765)}$	5.85 (0.584)	** 5.77 (0.531)**	4.43
Pakistan	5.64 (1.20)	$\underline{5.59\,(0.191)}$	**5.80 ( 0.062)**	5.76 (0.391)	4.431

Significant values are bold, underline, boldunderline.

**Table 12:** Model-Based GFR at Regional Level.

Region	Poisson	NB	ZIP	ZINB	Full Data
Punjab	**161.797 (2.191)**	$\underline{161.75\, (2.195)}$	170.62 (10.13)	172.12 (11.51)	160.784
Sindh	164.99 (2.46)	$\underline{{\textbf {164.33}}\,{\textbf {(2.48)}}}$	174.11 (10.20)	172.51 (8.75)	164.967
KPK	225.58 (50.89)	$\underline{{\textbf {174.95}}\,{\textbf {(2.64)}}}$	182.79 (8.33)	182.69 (8.369)	174.797
Bolachistan	$\underline{{\textbf {168.60}}\,{\textbf {(3.284)}}}$	169.31 (3.455)	171.639 (4.391)	172.448 (5.342)	168.600
ICT	${\textbf {181.956 (4.09)}}$	$\underline{181.47\,(4.38)}$	186.12 (8.04)	185.67 (7.65)	179.435
FATA	$\underline{{\textbf {153.197}}\,{\textbf {(3.635)}}}$	152.487 (3.66)	166.547 (13.856)	164.103 (12.113 )	153.163
Pakistan	$\underline{{\textbf {168.187}}\,{\textbf {(1.064)}}}$	167.459 (1.075)	176.005 (8.821)	174.751 (7.523)	168.165

Significant values are bold, underline, boldunderline.

**Table 13:** Model-Based GRR at Regional Level.

Region	Poisson	NB	ZIP	ZINB	FULL DATA
Punjab	2.162 ( 0.542)	**2.465 ( 0.052)**	2.528 (0.212)	2.553 (0.236)	2.704
Sindh	1.971 ( 0.504)	$\underline{{\textbf {2.515}}\,{\textbf {(0.057)}}}$	2.591 (0.184)	2.567 (0.161)	2.469
KPK	2.162 (0.475)	**2.507 (0.041)**	$\underline{2.563\,(0.173)}$	2.558 (0.168)	2.638
Bolachistan	2.162 ( 0.6552)	$\underline{2.618\,(0.1093)}$	**2.427 (0.0864)**	2.436 (0.0982)	2.8170
ICT	$\underline{1.971\,(0.368)}$	2.799 (0.302)	**2.813 (0.167)**	2.811 ( 0.1658)	2.3108
FATA	1.971 (0.834)	$\underline{2.496\,(0.342)}$	2.424 (0.219)	**2.388 (0.195)**	2.798
Pakistan	1.971 (0.444)	$\underline{{\textbf {2.540}}\,{\textbf {(0.078)}}}$	2.610 (0.026)	2.546 (0.152)	2.415

Significant values are bold, underline, boldunderline.

The model-based estimates on ASFR, TFR, GFR and GRR are obtained in this study using three responses (birth history during 1, 3 and 5 years preceding the interview). Interpretations are made on the basis of most suitable one (with smallest RMSE) from the estimates obtained under four different methods. The highest ASFR is estimated in Age group 25–29 and lowest is observed in 45-49 at national level. Same results for ASFR is obtained for Punjab, KPK and FATA. However, in Sindh, Balochistan and ICT highest ASFR is obtained for age group 20–24. The total TFR is estimated highest in ICT with 6.23 (about 6 births per woman) and lowest in Pubjab region with 5.45. TFR is estimated 5.8 (aorund 6 per woman during their whole reproductive age) at National level. The GFR is estimated highest in KPK with 175 per 1000 women and lowest in FATA with 153 per 1000 women. The GFR is estimated 168 per 1000 women at national level. Similarly, the GRR is obtained highest in ICT with 2.8 per married woman and lowest in Balochistan with 2.4.

## Conclusion

Estimation and inference of fertility rates posses severe challenges when surveys consist of missing responses on birth counts. Model-based approach by utilizing available data on related covariates aids in efficiency of estimated fertility rates. This article covers an etiological analysis of PDHS 2017-18 data on birth history to observe its significant determinants followed by construction of the model-based estimators on fertility rates including ASFR, TFR, GFR, and GRR. Estimation and inference of birth counts are considered under classical as well as Bayesian frameworks. The bootstrapped study reveals that the model-based estimators of fertility rates provide efficient results when relevant auxiliary data are available from the census at unit or cluster level. It is important to mention that the data about the majority of covariates considered for prediction in this study is not easy in practical situations at the individual level. However, data might be available for clusters through Civil Registration of Vital events (CRVE) or from previously conducted surveys. The predictive approach is suggested here in constructing rates in case of missing responses on birth and for small area estimation. However, in case of model failure the proposed estimation method would not work well and a more robust model assisted strategy is needed. Same authors are working on constructing the same rates using model assisted strategies.

## Appendix

See Figs. 4, 5, 6, 7, 8, 9, 10, 11, 12, 13, 14, and 15.
